# Genetic evaluation of Jatropha backcross hybrid clones (BC4F1) for yield and oil quality

**DOI:** 10.3389/fgene.2022.953486

**Published:** 2022-08-19

**Authors:** M. V. Jawahar Vishnu, K. T. Parthiban, M. Umadevi, R. Jude Sudhagar, C. Cinthia Fernandaz, Talha Javed, Uttam Kumar, Saqer S. Alotaibi

**Affiliations:** ^1^ Forest College and Research Institute, TNAU, Mettupalayam, India; ^2^ Center for Plant Breeding and Genetics, TNAU, Coimbatore, India; ^3^ Department of Agronomy, University of Agriculture, Faisalabad, Pakistan; ^4^ College of Plant Protection, Fujian Agriculture and Forestry University, Fuzhou, China; ^5^ Department of Biotechnology, College of Science, Taif University, Taif, Saudi Arabia

**Keywords:** Jatropha, genetic analysis, correlation, heritability, biochemical analysis

## Abstract

*Jatropha curcas* is a tropical species that has been recognized as a promising biodiesel plant. During 2018–2021, researchers at Forest College and Research Institute, Mettupalayam, elicited information on Jatropha’s biochemical characteristics, growth performance, variability, and association studies for biometric variables using five backcross (BC_4_F_1_) hybrid clones of Jatropha with a control variety TNMC 7. In terms of seed yield, two hybrid clones, CJH 13 (1,218.60 g) and CJH 12 (1,034.40 g), outperformed the other hybrid clones. The seed oil content was higher in CJH 5 (34.19%). The seed oil content had moderate PCV (16.49%) and GCV (16.39%) values, as well as high heritability (99%) and genetic advance (33.56%) as a percentage of the mean. The number of fruits per bunch (0.845 and 0.850) and the number of bunches per branch (0.771 and 0.788) had significant positive phenotypic and genotypic correlations with seed yield, respectively. The iodine numbers, cetane numbers, and saponification values of all hybrid clones were acceptable and satisfactory and were in good compliance with Indian and international biodiesel standards.

## Introduction

Automobile pollution, which causes climate change, has greatly accelerated environmental degradation. Numerous studies conducted in this area have focused on alternative fuels that can increase efficiency and reduce emissions (Seela et al., 2019). According to the British Petroleum statistical review report, nearly 80% of the world’s energy needs are met by fossil fuels. Between 2015 and 2019, fossil fuels accounted for an average of 85.16% of primary energy consumption; their consumption could reach 143 and 191 MT by 2025 and 2030, respectively, based on an increase of 6% per annum (Sayyed et al., 2022). India is a rapidly growing country with a population predicted to outgrow the rest of the world. India, as a country with limited fossil fuel sources, relies on imports of fossil fuels, the most common of which is crude oil. While rising fuel consumption has prompted a thorough assessment of alternate energy sources, fossil fuel reserves are being depleted (Witze, 2007). The government of India approved a National Policy on Biofuels in 2009, which established a non-mandatory target of 20% blending of biodiesel by 2017 (Bandyopadhay, 2015). India imports 40–50% of its edible oil for human use, making the utilization of edible oil resources for biofuel generation unfeasible. Under such circumstances, it is imperative to seek alternate, non-edible sources for biodiesel production.

In India, more than 100 species of tree-borne oilseeds have been identified as sources of biodiesel. Among these, the tropical physic nut tree (*Jatropha curcas*), a non-edible oil-bearing tree, has gained the attention of several development agencies and the Planning Commission of India. The genus Jatropha belongs to the family Euphorbiaceae, which originated in Central and South America and is distributed across tropical and subtropical regions of Africa and America. *J. curcas* is widely considered as a potential source for biodiesel production because of its short gestational period, small canopy suitable for high-density planting, convenience of seed collection, high seed oil content, ease of propagation through seeds and cuttings, drought hardiness, long productive period (40 years), rapid growth, resistance to animal grazing, productivity in both good and degraded soils, and widespread adaptability (Basha and Sujatha, 2007). The seeds are high in protein and are a key source of oil. The protein content of *J. curcas* seed meal has been shown to compare favorably to soybean meal, with a decent balance of essential amino acids (excluding lysine) (Basha and Sujatha, 2007).

*J. curcas* is valuable to consider because it does not compete with the food industry. The required amount of production is attainable, given that *J. curcas* has a productive life of roughly 50 years and reaches its peak productivity after only 4–5 years (Divakara et al., 2012). Additionally, this species has demonstrated the capacity to adapt to various agroclimatic situations, including infertile soils, making it appropriate for growing in damaged soils while guarding against erosion. These traits make the cultivation of *J. curcas* appropriate for conventional agriculture in marginal lands, where it could raise farmers’ socioeconomic standing and quality of life, especially in developing nations (Bekalu and Fekad, 2020).

Jatropha breeding programs are limited in comparison to other oil seed species such as soybean, cotton, peanut, sunflower, and castor, and there is inadequate research that precisely and scientifically authenticates the effect of field activity on the seed yield of *J. curcas* (Che Hamzah et al., 2020). Interspecific hybridization, in combination with backcross breeding, is a powerful tool for transferring a desired gene into a cultivar and can be used with a variety of plants. Interspecific hybridization with sexually compatible species facilitates the introgression of desirable characters, such as high oil content, oil quality, resistance to insect pests and diseases, reduced toxicity, and improved growth in problematic sites (Sujatha, 2020). Hence, Forest College and Research Institute (FC&RI) initiated an interspecific hybridization program using *J. curcas* as the female recipient; other Jatropha species, including *Jatropha integerrima*, *Jatropha podagrica*, *Jatropha villosa*, *Jatropha tanjorensis*, *Jatropha gossypifolia*, *Jatropha glandulifera*, *Jatropha multifeda*, and *Jatropha maheswari*, were used as pollen donors, resulting in novel hybrids with high production potential, root rot resistance, and frost tolerance. Among the several crossings, *J. curcas* and *J. integerrima* generated successful hybrids with higher seed sets, whereas others failed to produce seeds due to crossability barriers in the pre-zygotic or post-zygotic states (Parthiban et al., 2011). The F_1_ progeny of successful hybrids exhibited robust growth but had small fruit resembling *J. integerrima*. The resulting successful hybrid was backcrossed with *J. curcas* to produce offspring with distinct fruit, yield, and oil characteristics (Parthiban et al., 2009).

The most reliable and efficient method of evaluating genetic diversity is to study morphometric features in field trials. Because analyzed genetic differences are the best input for breeding and conservation efforts, field trial data are crucial (Eriksson et al., 1995). Since trees have high levels of variability, tree-improvement programs require comprehensive studies of variation. In Jatropha, such extensive research on backcross hybrids is lacking. Any crop-improvement program requires knowledge of existing genetic variability and of the relationships between various yield-related factors. In Jatropha, research on genetic variability and heritability in terms of growth and yield qualities is still in its early stages. Most traits of economic importance are complexly inherited, and the component characters show different types of associations with other traits. As a result, it is worthwhile to investigate the relationship between yield and yield related attributes, which indicated the yield contributing characters, for which systematic investigations in backcross Jatropha hybrids are insufficient.

Jatropha seed is higher in protein, carbohydrate, and lipid content than other seeds, and it is well known for its high fuel value. It has a 40–50 percent oil content. The chemical properties of oil (viz., acid number, free fatty acid content, iodine number, saponification value, and cetane number) directly influence the quality of oil for biodiesel production. However, such reports are inadequate in backcross hybrid clones of Jatropha. Against this backdrop, the current study investigated aspects of backcross (BC4F1) hybrid clones of Jatropha. It aimed 1) to evaluate BC4F1 clones for growth and yield attributes through clonal tests, 2) to analyze the genetic parameters and study the associations among the growth and yield attributes, and 3) to determine the oil content and oil quality of the promising clones.

## Materials and methods

The genus Jatropha belongs to the family Euphorbiaceae and contains 176 species, of which *J. curcas* L.—variously denoted as physic nut, ratanjot, or Barbados nut—is assumed to be the original form, from which other Jatropha species have evolved with variations in vegetative and reproductive characters (Dehgan and Webster, 1978). The plant is native to South America and Africa but was later distributed to other countries around the world by the Portuguese colonizers (Gübitz et al., 1999). Today, it is found in almost all the tropical and subtropical regions of the world. The botanist Carl Von Linne first classified the plant in 1753, giving it the botanical name “*Jatropha curcas*” from the Greek word “Jatros” (meaning a “Doctor”) and “trophe” (meaning “nutrition”). The plant is considered to be a shrub or small tree, with a height generally up to 5 m. The lifespan of the plant is up to 50 years (Azam et al., 2005). The shrub produces fruits in winter, when it is leafless, and it may produce several crops during the year if the soil moisture is good and temperatures are acceptable. Flowering in *J. curcas* depends on its locality and agroclimatic conditions. In Tamilnadu, flowering and fruiting occur nearly all year, but in North India, flowering generally occurs from August to December. Fruits mature within 2 months of flowering (Paramathma, 2010). Jatropha seeds may be harvested after 5 months of age, and the productivity is steady after 1 year of age. Jatropha seeds contain viscous oil, having seed oil content of 30–48.5%, which is a promising source of biodiesel (Parthiban et al., 2009).

**FIGURE 1:**
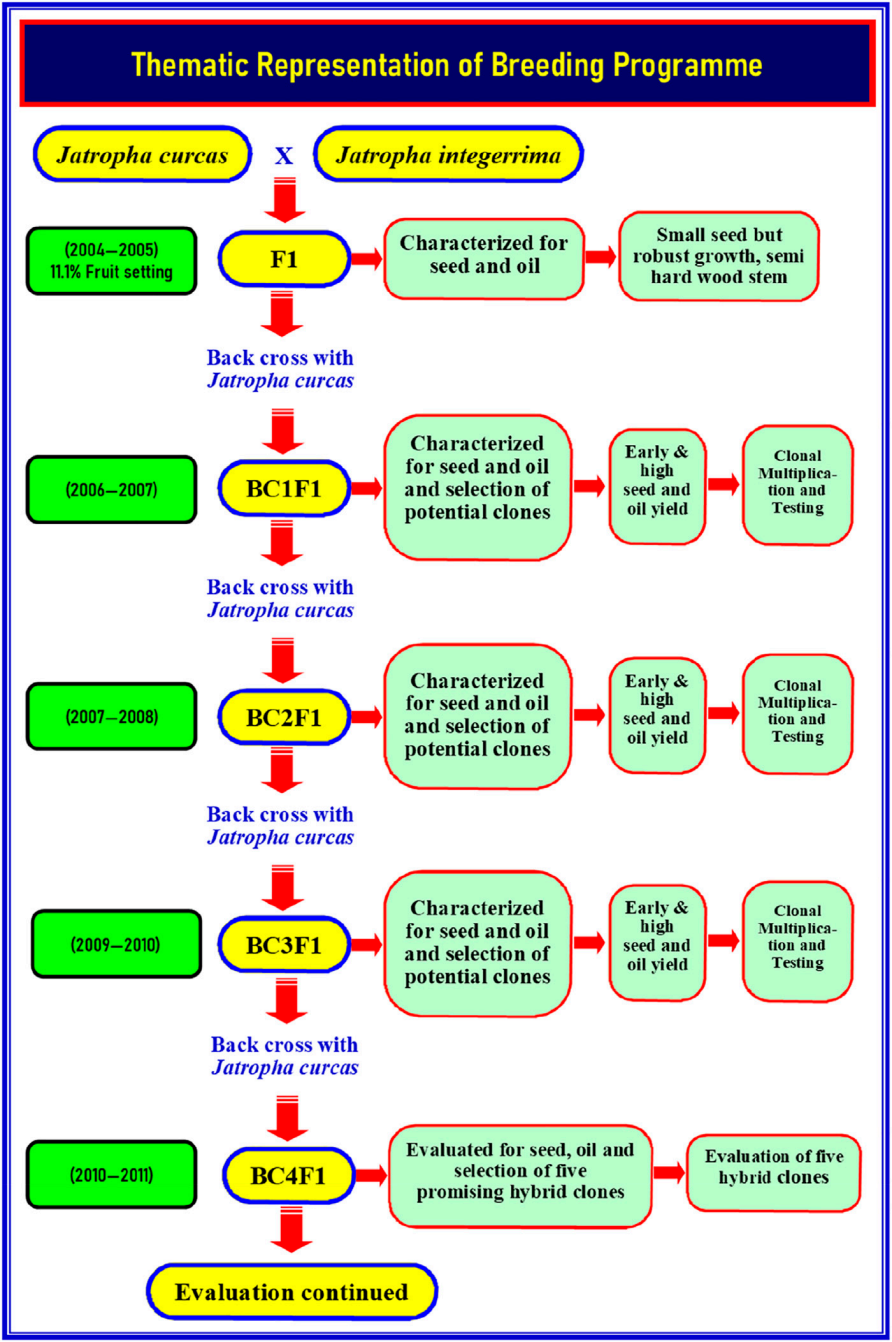
Thematic representation of breeding program.

The material for the present study consisted of five BC_4_F_1_ backcross hybrid clones of Jatropha: CJH 3, CJH 5, CJH 9, CJH 12, and CJH 13. The successful hybrid clones were developed through interspecific crosses between *J. curcas* and *J. integerrima*. The promising F_1_ plants (11.1% fruit setting with small fruit size) were then backcrossed with *J. curcas* (TNMC 7) to increase seed size (Parthiban et al., 2011). The resultant BC1F1 progenies were raised to the second generation and investigated for flowering and fruiting character. The superior hybrid clones of BC1F1 progenies (clones) were backcrossed with *J. curcas*, which resulted in BC2F1 hybrids. The superior hybrid clones of BC2F1 hybrids (clones) were further backcrossed with *J. curcas*, resulting in BC3F1 hybrids. The superior BC3F1 hybrid clones were backcrossed again with *J. curcas*, resulting in BC_4_F_1_ hybrids. From successive and continued back crossing (Figure 1), five BC_4_F_1_ backcross derivative clones were identified by their superior growth, seed, and oil yield. These were multiplied using apical shoot cuttings and are termed hybrid clones. The seeds were used to raise the first generation, and the first-generation apical shoot cuttings were used to develop clonal plantlets. The plantlets were deployed in field trials at FC&RI in Mettupalayam (11˚19′ N; 76˚56′ E; 300 m above MSL), along with the control variety (TNMC 7), in a randomized block design with four replications with 3 m × 3 m spacing. The hybrid clones were pruned once per year after fruiting season; the cumulative seed yield provided in the table represents 6–7 harvests per year.

The following observations (viz., plant height, basal diameter, sturdiness quotient, number of primary branches, number of secondary branches, number of male flowers, number of female flowers, male-female flower ratio, number of fruits per bunch, number of bunches per branch, seed yield per plant, hundred-fruit weight, fruit length, fruit width, fruit aspect ratio, hundred-seed weight, shelling percent, seed length, seed width, seed aspect ratio, kernel oil content, and seed oil content) were recorded for 3 years. The seed oil was extracted via solvent extraction, using hexane (40–60°C) as a solvent (A.O.A.C., 1975).

### Determination of the acid value and free fatty acid content

The acid number and free fatty acid content of oil were determined by the procedures of A.O.A.C. (1975):

$$\mathrm{Acid}\;value=\frac{V\times N\times 56.1}{W}$$

$$\mathrm{Free}\;fatty\;acid\;as\;oleic\;acid,per\;cent\;by\;weight=\frac{28.2\times V\times N}{W}$$

where

V = volume of KOH used.

N = normality of KOH.

W = weight of the sample (g).

### Determination of the saponification number

The saponification number was assessed per the method of A.O.A.C. (1975):

$$\mathrm{Saponification}\;number=\frac{\left(b-a\right)\times 0.02805\times 1000}{\text{Weight of the sample}}$$

where

b = ml of 0.5 N HCL required by the blank.

a = ml of 0.5 N HCL required by the sample.

### Determination of the iodine number

The iodine number was determined by the Hanus iodine method (A.O.A.C., 1975):

$$\mathrm{Iodine}\;number=\frac{\left(B-S\right)\times N\times 12.69}{\mathrm{Weight}\;of\;the\;sample}$$

where

B = ml of 0.1 N sodium thiosulfate required by blank.

S = ml of 0.1 N sodium thiosulfate required by sample.

N = normality of sodium thiosulfate solution.

### Determination of the cetane number

The cetane number of the Jatropha methyl esters was predicted per Krisnangkura (1986) :

Cetane number=46.3+(5458/SN−0.225∗IV)

where

SN = saponification number.

IV = iodine value.

### Statistical analysis

The data for growth performance, variability, association analysis, and biochemical features of seed oil were analyzed using one-way analysis of variance (ANOVA), and Duncan’s multiple range test was employed to compare treatment means. The data was analyzed using the IBM-SPSS analytical software program, version 20.0 (IBM Corporation, USA). Correlation coefficients were utilized to assess the relationships between the various variables in the experiment (F-test) (Goulden, 1936). The genotypic correlation coefficients were divided into direct and indirect effects using path coefficient analysis (Dewey and Lu, 1959).

## Results

### Variation in growth and yield attributes

Duncan’s multiple comparison study for growth performance differed significantly (*p* = 0.05) across different hybrid clones over 3 years, as shown in Tables 1 and 2.

**TABLE 1:** Mean performance in growth parameters of Jatropha hybrid clones.

Clones	Plant height (cm)	Basal diameter (cm)	Sturdiness quotient	No. of primary branches	No. of secondary branches	No. of male flowers	No. of female flowers	Male/female flower ratio	Fruits/bunch	Bunches/Branch	Cumulative seed yield (g)
First year (2018–2019)											
CJH 3	117.33 ± 5.51^b^	5.24 ± 0.06^ab^	22.39 ± 0.58^bc^	3.17 ± 0.03^d^	4.75 ± 0.02^a^	158.50 ± 8.23^c^	24.25 ± 0.15^ab^	6.54 ± 0.22^c^	15.20 ± 0.60^b^	4.25 ± 0.16^ab^	353.29 ± 17.75^d^
CJH 5	112.44 ± 5.82^b^	5.07 ± 0.21^ab^	22.18 ± 0.56^bc^	3.11 ± 0.01^d^	4.52 ± 0.15^a^	172.25 ± 2.28^c^	26.85 ± 0.62^a^	6.42 ± 0.22^c^	16.50 ± 0.00^ab^	4.00 ± 0.09^b^	381.40 ± 18.90^cd^
CJH 9	108.44 ± 0.50^b^	5.48 ± 0.17^a^	19.79 ± 0.23^c^	4.12 ± 0.15^c^	3.95 ± 0.19^b^	169.25 ± 2.60^c^	26.65 ± 0.95^a^	6.35 ± 0.30^c^	16.40 ± 0.62^b^	4.75 ± 0.24^a^	397.23 ± 3.00^bc^
CJH 12	116.56 ± 3.42^b^	5.00 ± 0.12^ab^	23.31 ± 1.18^b^	4.70 ± 0.04^a^	4.35 ± 0.12^ab^	196.75 ± 9.06^b^	24.50 ± 1.23^ab^	8.03 ± 0.27^b^	18.35 ± 0.90^a^	4.75 ± 0.24^a^	426.90 ± 0.11^b^
CJH 13	132.22 ± 1.92^a^	5.03 ± 0.22^ab^	26.29 ± 1.31^a^	4.33 ± 0.01^b^	4.61 ± 0.13^a^	192.25 ± 2.74^b^	22.25 ± 1.03^b^	8.64 ± 0.07^b^	18.35 ± 0.63^a^	4.25 ± 0.01^ab^	484.40 ± 13.05^a^
TNMC 7 (Control)	106.11 ± 3.73^b^	4.72 ± 0.11^b^	22.48 ± 0.37^b^	2.55 ± 0.03^e^	3.20 ± 0.16^c^	225.50 ± 6.00^a^	18.25 ± 0.94^c^	12.35 ± 0.40^a^	12.90 ± 0.24^c^	4.00 ± 0.01^b^	297.40 ± 4.76^e^
*p* value	0.007^ns^	0.85^ns^	0.003^ns^	<0.0001^a^	<0.0001^a^	<0.0001^a^	<0.0001^a^	<0.0001^a^	<0.0001^a^	0.14^ns^	<0.0001^a^
Second year (2019–2020)											
CJH 3	121.24 ± 5.10^ab^	12.40 ± 0.09^bc^	9.78 ± 0.31^b^	7.17 ± 0.05^b^	6.75 ± 0.07^a^	233.25 ± 4.79 ^b^	34.50 ± 0.22^bc^	6.80 ± 0.02^bb^	21.40 ± 0.91^a^	6.25 ± 0.30^ab^	827.65 ± 29.64^b^
CJH 5	120.63 ± 1.48^ab^	13.98 ± 0.54^a^	8.6 ± 0.35^c^	6.11 ± 0.32^c^	7.52 ± 0.26^bcd^	227.75 ± 6.09 ^b^	36.95 ± 1.89^c^	6.20 ± 0.20^cd^	22.35 ± 0.98^a^	6.00 ± 0.10^b^	819.74 ± 26.33^b^
CJH 9	118.27 ± 0.20^b^	12.74 ± 0.19^b^	9.28 ± 0.06^bc^	9.12 ± 0.02^a^	9.95 ± 0.49^a^	217.75 ± 1.46 ^b^	36.89 ± 1.88^c^	5.90 ± 0.16^d^	21.35 ± 0.90^a^	6.75 ± 0.02^a^	887.43 ± 11.47^ab^
CJH 12	116.70 ± 5.41^b^	12.40 ± 0.25^bc^	9.41 ± 0.44^bc^	7.70 ± 0.12^b^	8.35 ± 0.00^b^	213.25 ± 4.35 ^b^	34.51 ± 1.07^bc^	6.20 ± 0.14^cd^	22.35 ± 0.05^a^	6.75 ± 0.25^a^	913.50 ± 46.60^a^
CJH 13	131.97 ± 1.18^a^	11.23 ± 0.47^c^	11.75 ± 0.22^a^	7.33 ± 0.21^b^	7.61 ± 0.15^bc^	216.75 ± 9.86^b^	32.51 ± 0.29^b^	6.70 ± 0.31^bc^	23.35 ± 1.14^a^	6.25 ± 0.19^ab^	934.58 ± 5.22^a^
TNMC 7 (Control)	109.70 ± 4.02^b^	11.49 ± 0.46^c^	9.55 ± 0.43^bc^	6.55 ± 0.16^cc^	7.20 ± 0.26^cd^	281.75 ± 8.59^a^	28.70 ± 0.65^c^	9.80 ± 0.03^a^	16.90 ± 0.85^b^	6.00 ± 0.04^b^	526.12 ± 9.02^c^
*p* value	0.19^ns^	0.003^ns^	<0.0001^a^	<0.0001^a^	<0.0001^a^	<0.0001^a^	0.004^ns^	<0.0001^a^	0.003^ns^	0.37^ns^	<0.0001^a^
Third year (2020–2021)											
CJH 3	125.15 ± 2.63^abc^	19.44 ± 0.96^b^	6.44 ± 0.08^bc^	7.15 ± 0.04^c^	9.50 ± 0.08^a^	245.75 ± 4.94^b^	35.50 ± 0.59^ab^	6.92 ± 0.12^b^	22.50 ± 0.39^b^	7.20 ± 0.17^a^	924.80 ± 17.72^cd^
CJH 5	128.83 ± 3.82^ab^	22.78 ± 0.48^a^	5.66 ± 0.10^d^	7.12 ± 0.05^c^	9.62 ± 0.06^a^	252.50 ± 10.56^b^	38.75 ± 1.78^a^	6.52 ± 0.10^bc^	22.25 ± 0.36^b^	6.50 ± 0.23^b^	859.70 ± 22.48^d^
CJH 9	128.10 ± 4.51^ab^	19.78 ± 0.22^b^	6.48 ± 0.20^b^	9.50 ± 0.16^a^	9.85 ± 0.45^a^	237.25 ± 10.69^b^	34.25 ± 0.66^b^	6.93 ± 0.02^b^	22.25 ± 0.49^b^	6.20 ± 0.17^b^	950.90 ± 33.68^c^
CJH 12	116.85 ± 5.34^bc^	19.89 ± 0.39^b^	5.87 ± 0.26^d^	8.42 ± 0.05^b^	9.25 ± 0.18^a^	226.75 ± 7.54^b^	36.50 ± 1.56^ab^	6.21 ± 0.19^c^	23.50 ± 1.20^b^	7.50 ± 0.11^a^	1,034.40 ± 33.47^b^
CJH 13	131.72 ± 6.09^a^	18.11 ± 0.26^b^	7.27 ± 0.15^a^	9.52 ± 0.31^a^	9.60 ± 0.22^a^	238.25 ± 8.98^b^	36.50 ± 1.49^ab^	6.53 ± 0.03^bc^	26.25 ± 0.84^a^	7.50 ± 0.21^a^	1,218.60 ± 21.91^a^
TNMC 7 (Control)	113.30 ± 0.67^c^	19.11 ± 0.91^b^	5.93 ± 0.15^cd^	7.50 ± 0.37^c^	8.25 ± 0.25^b^	316.50 ± 10.12^a^	29.50 ± 1.15^c^	10.73 ± 0.29^a^	17.50 ± 0.19^c^	6.00 ± 0.13^b^	653.66 ± 16.12^e^
*p* value	0.57^ns^	0.004^ns^	<0.0001^a^	<0.0001^a^	0.007^ns^	<0.0001^a^	0.005^ns^	<0.0001^a^	<0.0001^a^	<0.0001^a^	<0.0001^a^

a Highly significant difference at *p* < 0.001 level of probability, and ns - no significance. Values with different superscripts were significantly different at *p* < 0.05 level of probability.

**TABLE 2:** Mean performance in fruit and seed attributes of Jatropha hybrid clones.

Clones	100 Fruit weight (g)	Fruit length (cm)	Fruit width (cm)	Fruit aspect ratio	100 seed weight (g)	Seed length (cm)	Seed width (cm)	Seed aspect ratio	Shelling percent	Seed oil content (%)	Kernel oil content (%)
CJH 3	112.57 ± 0.74^a^	2.00 ± 0.04^b^	1.57 ± 0.04^c^	1.28 ± 0.02^ab^	59.37 ± 0.52^abc^	1.73 ± 0.00^a^	0.79 ± 0.03^a^	2.19 ± 0.02^ab^	61.57 ± 2.13^a^	27.93 ± 0.00^b^	54.80 ± 0.80^a^
CJH 5	110.68 ± 2.39^a^	2.10 ± 0.08^b^	1.69 ± 0.01^abc^	1.26 ± 0.00^ab^	57.48 ± 0.45^bc^	1.72 ± 0.06^a^	0.80 ± 0.00^a^	2.15 ± 0.05^ab^	61.96 ± 1.24^a^	34.19 ± 0.37^a^	53.60 ± 1.96^a^
CJH 9	112.66 ± 0.13^a^	2.12 ± 0.07^b^	1.63 ± 0.05^bc^	1.33 ± 0.05^a^	59.46 ± 1.93^abc^	1.74 ± 0.00^a^	0.80 ± 0.02^a^	2.18 ± 0.03^ab^	60.43 ± 2.50^a^	23.74 ± 0.58^d^	43.50 ± 0.17^b^
CJH 12	114.40 ± 2.32^a^	2.05 ± 0.09^b^	1.74 ± 0.01^ab^	1.18 ± 0.05^b^	61.20 ± 1.46^ab^	1.68 ± 0.07^a^	0.78 ± 0.00^a^	2.17 ± 0.09^ab^	62.22 ± 1.32^a^	25.76 ± 0.38^c^	45.40 ± 2.10^b^
CJH 13	116.81 ± 4.36^a^	2.16 ± 0.02^b^	1.78 ± 0.07^ab^	1.22 ± 0.05^ab^	63.61 ± 1.70^a^	1.71 ± 0.02^a^	0.81 ± 0.03^a^	2.12 ± 0.07^b^	58.99 ± 0.45^a^	24.64 ± 0.54^cd^	53.20 ± 2.55^a^
TNMC 7 (Control)	109.56 ± 2.29^a^	2.37 ± 0.02^a^	1.80 ± 0.05^a^	1.33 ± 0.00^a^	56.36 ± 1.06^c^	1.75 ± 0.06^a^	0.75 ± 0.00^a^	2.34 ± 0.06^a^	63.85 ± 0.58^a^	21.92 ± 0.62^e^	35.40 ± 0.26^c^
*p* value	0.399^ns^	0.024^ns^	0.030^ns^	0.135^ns^	0.025^ns^	0.939^ns^	0.624^ns^	0.628^ns^	0.401^ns^	<0.0001^a^	<0.0001^a^

a Highly significant difference at *p* < 0.001 level of probability, and ns - no significance. Values with different superscripts were significantly different at *p* < 0.05 level of probability.

The mean performance of 5 Jatropha hybrid clones, along with the control variety, in their growth and yield attributes is presented in Table 1. The hybrid clone CJH 13 exhibited superiority in characteristics including plant height (131.72 ± 6.09 cm) and the number of fruits per bunch (26.25 ± 0.84). The number of fruit bunches per branch was higher in CJH 12 and CJH 13. The seed yield from the hybrid clones collected for the third year (7 harvests) varied significantly and ranged from 1,218.60 ± 21.91 g (CJH 13) to 653.66 ± 16.12 g (TNMC 7).

### Variability in fruit and seed characters

CJH 13 exhibited superiority in terms of hundred-fruit weight (116.81 ± 4.36 g) and hundred-seed weight (63.61 ± 1.70 g), whereas TNMC 7 showed an advantage in terms of fruit length, fruit width, and shelling percent. Seed oil content varied between 34.19 ± 0.37 percent (CJH 5) and 21.92 ± 0.62 percent (TNMC 7). The kernel oil content ranged between 54.80 ± 0.80 percent (CJH 3) and 35.40 ± 0.26 percent (TNMC 7).

### Genetic estimates of growth, yield, and yield-contributing characters

The male–female flower ratio demonstrated moderate PCV (21.22%) and GCV (21.10%), followed by seed yield, number of primary branches, number of secondary branches, number of male flowers, and number of fruits per bunch (Table 3). All the traits recorded high heritability (>60%). The genetic advance as percent over the mean was moderate for the male–female flower ratio (43.22%) and seed yield (32.85%). Among the fruit’s physical attributes, fruit yield exhibited moderate PCV (19.14%) and GCV (19.11%) values. All traits recorded high heritability (>60%), except for fruit aspect ratio (31%) and fruit width (54%). Fruit yield (39.32%) alone recorded high genetic advance as a percentage of mean compared to other parameters. Among the seed parameters, seed oil content registered moderate PCV (16.49%) and GCV (16.39%) values. All parameters recorded high heritability (>60%), except for seed length (43%) and seed width (46%). Seed oil content (33.56) alone recorded high genetic advance as a percentage of mean (Table 3).

**TABLE 3:** Genetic estimates of hybrid clones for biometrical, fruit, and seed characters.

Traits	PCV %	GCV %	Heritability (%)	GA (%) of mean
Plant height	6.08	5.83	92.00	11.50
Basal diameter	5.24	4.96	89.00	9.67
Sturdiness quotient	5.18	4.64	80.00	8.56
No. of primary branches	14.71	14.65	95.00	30.03
No. of secondary branches	14.45	14.41	95.00	29.59
No. of male flowers	11.14	11.03	94.00	22.49
No. of female flowers	9.13	9.09	95.00	18.64
F:M ratio	21.22	21.10	95.00	43.22
No. of fruits/bunch	10.78	10.73	95.00	22.01
No. of bunches/branch	5.60	5.48	93.00	11.05
Seed yield	16.05	16.00	95.00	32.85
Hundred-fruit weight	2.50	2.23	80.00	4.11
Fruit length	6.41	5.96	86.00	11.41
Fruit width	6.54	4.80	54.00	7.27
Fruit aspect ratio	6.86	3.80	31.00	4.33
Fruit yield	19.14	19.11	95.00	39.32
Hundred-seed weight	4.72	4.22	80.00	7.77
Seed length	1.88	1.23	43.00	1.67
Seed width	3.80	2.57	46.00	3.60
Seed aspect ratio	3.84	3.44	80.00	6.35
Shelling percent	3.06	2.55	70.00	4.38
Seed oil content	16.49	16.39	95.00	33.56

### Association between yield and yield-contributing characters

Highly significant and positive phenotypic and genotypic correlations were recorded between the number of fruits per bunch (0.845 and 0.850) and the number of bunches per branch (0.771 and 0.788) on seed yield, followed by the number of female flowers (0.555 and 0.557), respectively (Table 4). Significant and negative phenotypic and genotypic correlations were recorded between the number of male flowers (–0.843 and –0.852) and male–female flower ratio (–0.760 and –0.765) on seed yield, respectively. The number of female flowers was positively correlated with the number of fruits per bunch (0.739 and 0.745) (Table 4).

**TABLE 4:** Phenotypic and genotypic correlation coefficients among biometrical traits on seed yield.

Traits		Plant height	Basal diameter	Sturdiness quotient	Number of primary branches	Number of secondary branches	Number of male flowers	Number of female flowers	Male female flower ratio	Number of fruits/bunch	Number of bunches/branch	Seed yield
Plant height	P	1.000	0.581	0.599	0.183	0.181	–0.598	0.585	–0.639	0.536	–0.050	0.149
	G	1.000	0.634	0.595	0.207	0.182	–0.647	0.611	–0.678	0.558	–0.027	0.161
Basal diameter	P		1.000	–0.304	0.689	0.557	–0.611	0.759^a^	–0.713	0.459	0.535	0.322
	G		1.000	–0.244	0.729	0.594	–0.654	0.815^a^	–0.764^a^	0.483	0.566	0.341
Sturdiness quotient	P			1.000	–0.456	–0.325	–0.095	–0.065	–0.041	0.171	–0.581	-0.144
	G			1.000	–0.492	–0.378	–0.129	–0.084	–0.052	0.191	–0.621	-0.154
No. of primary branches	P				1.000	0.840^a^	–0.517	0.373	–0.468	0.277	0.864^a^	0.502
	G				1.000	0.847^a^	0.523	0.381	–0.475	0.278	0.883^a^	0.504
No. of secondary branches	P					1.000	–0.433	0.467	–0.441	0.227	0.726	0.494
	G					1.000	–0.441	0.470	–0.446	0.224	0.747^a^	0.497
No. of male flowers	P						1.000	–0.761^a^	0.959^a^	–0.947^a^	–0.598	–0.843^a^
	G						1.000	–0.768^a^	0.960^a^	–0.960^a^	–0.631	–0.852^a^
No. of female flowers	P							1.000	–0.911^a^	0.739^a^	0.419	0.555
	G							1.000	–0.914^a^	0.745^a^	0.436	0.557
Male-female flower ratio	P								1.000	–0.922^a^	–0.541	–0.760^a^
	G								1.000	–0.932^a^	–0.569	–0.765^a^
No. of fruits/bunch	P									1.000	0.476	0.845^a^
	G									1.000	0.487	0.850^a^
No. of bunches/branch	P										1.000	0.771^a^
	G										1.000	0.788^a^
Seed yield	P											1.000
	G											1.000

a Significant at 5% level (F test).

At both the phenotypic and genotypic levels, correlation studies found that hundred-fruit weight (0.540 and 0.605) had a positive relationship with fruit output. Except for fruit length (–0.720) and fruit aspect ratio (–0.838), which had significant negative correlations with fruit yield at the genotypic level, all other fruit parameters were negative and non-significant (Table 5). At both the phenotypic and genotypic levels, seed width (0.325 and 0.464) had a positive connection with seed oil content. The link between seed oil content and all other seed parameters was negative and non-significant (Table 6).

**TABLE 5:** Phenotypic and genotypic correlation coefficients among fruit attributes on fruit yield.

Traits		Hundred-fruit weight	Fruit length	Fruit width	Fruit aspect ratio	Fruit yield
Hundred -fruit weight	P	1.000	−0.408	0.157	−0.540	0.540
	G	1.000	−0.428	0.048	−0.773^a^	0.605
Fruit length	P		1.000	0.548	0.342	−0.672
	G		1.000	0.800^a^	0.563	−0.720^a^
Fruit width	P			1.000	−0.454	−0.144
	G			1.000	−0.159	−0.205
Fruit aspect ratio	P				1.000	−0.459
	G				1.000	−0.838^a^
Fruit yield	P					1.000
	G					1.000

a Significant at 5% level (F test).

**TABLE 6:** Phenotypic and genotypic correlation coefficients among seed attributes on seed oil content.

Traits		Hundred- seed weight	Seed length	Seed width	Seed aspect ratio	Shelling percent	Seed oil content
Hundred- seed weight	P	1.000	−0.532	0.549	−0.721^a^	−0.651	−0.155
	G	1.000	−0.599	0.679	−0.760^a^	−0.878^a^	−0.188
Seed length	P		1.000	−0.225	0.434	0.200	−0.224
	G		1.000	−0.280	0.732^a^	0.135	−0.341
Seed width	P			1.000	−0.780^a^	−0.646	0.325
	G			1.000	−0.985^a^	−0.895^a^	0.464
Seed aspect ratio	P				1.000	0.649	−0.444
	G				1.000	0.865^a^	−0.471
Shelling percent	P					1.000	−0.002
	G					1.000	−0.002
Seed oil content	P						1.000
	G						1.000

a Significant at 5% level (F test).

### Biochemical properties of Jatropha hybrid clones

The acid value ranged between 7.05 ± 0.20 (CJH 12) and 5.18 ± 0.00 (TNMC 7). Free fatty acid content differed significantly among the different hybrid clones, ranging from 3.54 ± 0.16 percent (CJH 12) to 2.60 ± 0.05 percent (TNMC 7). Saponification values ranged between 203.36 ± 5.35 (CJH 12) and 201.12 ± 1.85 (TNMC 7). Iodine value extended from 116.64 ± 1.93 (CJH 13) to 107.56 ± 3.39 (CJH 3). The hybrid clones varied from 49.20 ± 0.69 (CJH 3 and TNMC 7) to 47.12 ± 0.18 (CJH 13) in terms of the cetane number (Table 7).

**TABLE 7:** Biochemical Properties of Jatropha hybrid clones.

Clones	Acid value	Free fatty acid content (%)	Saponification value	Iodine value	Cetane number
CJH 3	5.27 ± 0.05^d^	2.65 ± 0.05^d^	201.40 ± 1.47^a^	107.56 ± 0.03^a^	49.20 ± 0.69^a^
CJH 5	5.60 ± 0.06^cd^	2.81 ± 0.20^cd^	201.96 ±6.97^a^	110.59 ± 5.52^a^	48.44 ± 1.27^a^
CJH 9	6.25 ± 0.15^b^	3.14 ± 0.00^b^	202.24 ± 9.68^a^	114.26 ± 1.72^a^	47.58 ± 0.70^a^
CJH 12	7.05 ± 0.20^a^	3.54 ± 0.16^a^	203.36 ± 5.35^a^	114.80 ± 3.91^a^	47.31 ± 2.28^a^
CJH 13	5.78 ± 0.19^c^	2.90 ± 0.02^c^	201.68 ± 3.27^a^	116.64 ± 1.93^a^	47.12 ± 0.18^a^
TNMC 7 (control)	5.18 ± 0.00^d^	2.60 ± 0.05^d^	201.12 ± 1.85^a^	107.74 ± 3.39^a^	49.20 ± 1.21^a^
*p* value	<0.0001^a^	<0.0001^a^	1.000^ns^	0.296^ns^	0.728^ns^

a Highly significant difference at *p* < 0.001 level of probability, and ns—no significance. Values with different superscripts were significantly different at *p* < 0.05 level of probability.

## Discussion

### Variation in growth and yield attributes

Through selection and further deployment in evaluation programs, any tree-improvement program utilizes existing genetic variability (Chaturvedi and Pandey, 2001). In this study, the plant height, basal diameter, sturdiness quotient, number of primary branches, number of secondary branches, number of male flowers, number of female flowers, male–female flower ratio, number of fruits per bunch, and number of bunches per branch all changed considerably under field settings. For various growth characteristics, the hybrid clones CJH 13 and CJH 5 recorded much higher values. Paramathma (2010); Maurya *et al.* (2013); Subashini *et al.* (2014); and Gawali *et al.* (2013) found comparable and considerable variance in growth indices in *J. curcas.*

*J. curcas* is a monoecious tree with a minimal number of female flowers relative to its numerous male flowers, which is the key reason for its low yield. Increasing the number of female flowers in the inflorescence may result in increased fruit set and, in turn, increased seed yield. In the present study, CJH 5 demonstrated superiority in terms of the number of female flowers (38.75) and registered a lower male–female flower ratio (6.52) compared to the other hybrid clones. Similar noteworthy studies on the floral characters of *J. curcas* were previously conducted by Paramathma (2010); Maurya *et al.* (2013) and Subashini *et al.* (2014) and lend support to the current investigation.

The seed yield of a tree is influenced by biometric traits (viz., number of branches, crown diameter, and age). In the present investigation, two hybrid clones, CJH 13 (1,218.60 g) and CJH 12 (1,034.40 g), showed significant performance for seed yield per plant at early stages of growth and development. These two hybrid clones are prominent, and the consistency and fidelity expressed by these hybrid clones could be useful for future improvement programs. Parthiban *et al.* (2011) observed that the fruit yield of *Jatropha* hybrid clones ranges from 131.78 to 857.51 g per plant. Divakara *et al.* (2012), in the case of *Pongamia pinnata*, also reported that not only plant growth characters but also environmental factors have vital roles in seed yield. Biabani *et al.* (2012) evaluated different Jatropha populations and reported that the seed yield per plant was highest in Indonesia, which recorded 46, 399, and 222 (g) for the first, second, and average years’ harvests, respectively. Indonesia was closely followed by the Philippines, with values of 35 (g) for the first year, and Malaysia, with values of 262 and 146 (g) for the second and average years’ harvests, respectively.

### Variability in fruit and seed characters

Nature offers plants with enormous energy potential. Jatropha is one of the most significant species that can be utilized in the form of biodiesel. The basic aim for any tree-breeding program is to exploit the available natural variability within a species, ultimately resulting in higher production in terms of yield. In the present investigation, significant differences were observed in the fruit’s physical traits (viz., fruit length, fruit width, fruit aspect ratio, and hundred-fruit weight). The hybrid clone CJH 13 recorded the highest significant values for hundred-fruit weight (116.81 g), whereas TNMC 7 exhibited superiority in terms of fruit length (2.37 cm), fruit width (1.80 cm), and fruit aspect ratio (1.33). Similar variability in the physical fruit parameters of *J. curcas* was previously recorded (Gawali et al., 2013), affirming the findings of the current study.

The study of seed parameters, along with oil content, is often considered to be a suitable step in the study of genetic variability in case of tree-borne oil seeds. Thus, seeds from trees with higher seed weights and oil content may be used for further improvement programs (Kaushik et al., 2007). The hybrid clone CJH 13 recorded the highest significant values for seed width (0.81 cm) and hundred-seed weight (63.61 g), CJH 5 registered the highest significant value for seed oil content (34.19%), and CJH 3 recorded high kernel oil content (54.80%). Comparable findings in *J. curcas* were previously reported by Tripathi *et al.* (2015) and Singh *et al.* (2016), which supports the results of present study.

### Genetic estimates of growth, yield, and yield-contributing characters

Variation describes noticeable changes across individuals for a specific trait. These differences are partially due to genetic control and partially due to the influence of the environment. All the growth attributes under study exhibited higher heritability values (0.80–0.99), suggesting that these traits are under strong genetic control. Lower heritability for vegetative characters and moderate-to-high heritability for reproductive characters have been reported in backcross populations of Jatropha (Subashini et al., 2014). There were high significant variations among the populations with regard to the yield and yield components, and results of ANOVA on these traits indicated highly significant variation among populations for first, second, and average years of harvesting (Biabani et al., 2012). Ghosh and Singh (2011) identified similar phenotypic variations in seed length, seed width, hundred-seed weight, oil content, seed yield, oil content percentage, and seed oil yield among *J. curcas* populations. Traits such as the number of branches, number of female flowers, number of fruits per bunch, and seed yield exhibited higher heritability (0.99) with moderate genetic advance; hence, these traits are under strong genetic control. The current findings corroborate earlier studies in Jatropha (Reeja, 2018), suggesting that the traits involved in high heritability with moderate genetic gain may be due to the presence of additive genes.

Phenotypic correlation is a product of the interaction between genotype and environment, whereas genotypic correlation is an assessed value. The complexity of seed yield is mostly influenced by the quantity of characteristics, which in turn affects the production of oil. In order to create a valuable and feasible breeding program for increasing seed yield, knowledge of the associations between these traits would be very supportive.

Fruit yield recorded high PCV (19.14) and GCV (19.11), and high heritability (0.99) and maximum genetic advance (39.32), followed by fruit length and hundred-fruit weight. Hence, this parameter could be used as a reliable trait for selection. The hundred-fruit weight, fruit length, fruit width, and fruit aspect ratio recorded low PCV and GCV, which agrees with the result of Gawali et al. (2013) in *J. curcas.* The seed oil content demonstrated higher phenotypic (16.49) and genotypic (16.39) coefficients of variation. The seed aspect ratio, seed length, seed width, shelling percent, and hundred-seed weight documented low PCV and GCV. Similar results have been observed in *J. curcas* (Gawali et al., 2013) and in backcross populations of Jatropha (Subashini et al., 2014), which corroborates the current findings. In the present investigation, seed oil content expressed high heritability (0.99), which indicated additive gene action and moderate heritability for seed length and seed width, showing that they are governed partly by additive gene actions. The distinctly higher heritability can be regarded as a key feature for Jatropha improvement because of its strong genetic control.

### Association between yield and yield-contributing characters

Correlation studies are important in determining the suitability of numerous traits for selection since selection of particular traits may bring desirable or undesirable changes in the related traits. For all the traits investigated, the genotypic correlation coefficient was higher than the corresponding phenotypic correlation coefficient, indicating that in the majority of cases, the environment has not appreciably influenced the associated traits. The number of fruits per bunch and the number of bunches per branch showed positive and significant correlations with seed yield, whereas the number of male flowers and male–female flower ratio showed negative and significant correlations with seed yield. Such positive and highly significant correlations in Jatropha can be used for further breeding programs. Comparable positive and significant correlations have been observed by Subashini *et al.* (2014) and Parthiban *et al.* (2011) in *J. curcas* and by Chauhan *et al.* (2005) in neem, which supports the results of current study. In addition, the basal diameter, sturdiness quotient, number of primary branches, number of secondary branches, number of male flowers, number of female flowers, male–female flower ratio, number of fruits per bunch, and number of bunches per branch showed strong correlation among themselves, signifying that some genes governing these characters may be closely associated with each other.

The number of male flowers, male–female flower ratio, number of fruits per bunch, and number of bunches per branch could serve as good predictors of seed yield in Jatropha*.* The traits of fruit length and fruit aspect ratio showed negative and significant correlations with fruit yield at the genotypic level only. Comparable results have been documented in *J. curcas* (Gawali et al., 2013; Singh et al., 2013). Seed width registered positive but non-significant correlation with seed oil content, which indicated that this parameter has contributed significantly to seed oil content and hence could act as a reliable index in Jatropha improvement programs. In contrast, all other seed parameters registered negative and non-significant correlations with seed oil content, which specified that these characters showed independent genetic control.

### Biochemical properties of Jatropha hybrid clones

Chemical properties are the vital properties that determine the present condition of oil (Nzikou et al., 2009). Several chemical properties of biodiesel permit it to burn cleanly and improve the combustion of diesel in blends. Some of the primary chemical properties which determine the suitability of oils for the utilization of biodiesel are acid value, iodine value, cetane number, free fatty acid content, and saponification value. Standards for these properties have been established worldwide and in India; the Bureau of Indian Standards has specified standards (IS:15,607, 2006). In this study, significant variations were documented for acid value of the seed oil of Jatropha hybrid clones. All the five hybrid clones recorded significantly higher acid values compared to the control variety, which was still higher than the standard value (0.5).

The properties of triglycerides and biofuels depend upon the degree of fatty acids (unsaturated and saturated) present in the molecules. Free fatty acid percentage differed significantly among the different hybrid clones deployed in the current investigation. The free fatty acid percentage ranged from 3.54% (CJH 12) to 2.60% (TNMC 7), and the increase in free fatty acid content could be due to oil storage. High free fatty acid content (>1%) results in soap formation and hence difficulty in the splitting of products, leading to low recovery of biodiesel (Crabbe et al., 2001). The free fatty acid and moisture content influence the transesterification of glycerides with alcohol using a catalyst. High free fatty acid percentage (2.23%) has been recorded earlier in Jatropha oil seeds (Goodrum, 2002).

Saponification value is an indicator of the extent of fatty acids present and rests on the molecular weight and percentage concentration of fatty acid components in the oil. Apart from biodiesel purposes, a high saponification value indicates that oils can be used in the production of liquid soap and shampoo. Only one hybrid clone (viz., CJH 12 (203.36)) recorded significantly higher saponification values compared to the control variety TNMC 7 (201.12). Similar studies on the saponification value of *J. curcas* hybrids have been reported by Ntaganda (2014).

The iodine value reflects the degree of the unsaturation of fats and oils. Higher iodine values indicate higher unsaturation of fats and oils (Knothe, 2002). The iodine value ranged from 116.64 (CJH 13) to 107.56 (CJH 3). These values indicate that the oils of hybrid clones are well below the limit of 115 set by the European standards (EN:14,214, 2008). Hence, all the hybrid clones deployed in the current investigation are qualified to produce biodiesel with respect to iodine value. The iodine values of Jatropha lie in the semi-drying oil group. Jatropha’s higher iodine value is due to the presence of high amounts of unsaturated fatty acids, such as oleic acid and linoleic acid.

The ignition quality of a fuel can be inferred through its cetane number (Azam et al., 2005). A fuel with high ignition quality also has a high cetane number, in which the ignition delay period between the start of fuel injection and the onset of auto ignition is short. These fuel properties are significant in deciding diesel engine operating features such as fuel conversion efficiency, smoothness of operation, misfire, smoke emissions, noise, and ease of starting. The cetane value was found to be significantly higher for CJH 3 and TNMC 7 (49.20), which is near the acceptable minimum standard value of 51, per the IS:15,607 (2006) biodiesel specification of the Bureau of Indian Standards. The minimum acceptable cetane number, per the American standards for biodiesel, is 47, and most of the hybrid clones recorded significantly higher cetane numbers compared to the American standard (ASTM D6751, 2002). All the hybrid clones deployed in the current investigation recorded cetane numbers in the range of 47–49, indicating their usability as biodiesel and substantiating the study of Sivaramakrishnan and Ravikumar (2012).

## Conclusion

Through a holistic analysis, the current study identified the superiority of two hybrid clones (viz., CJH 13 and CJH 12) for productivity of seeds and one hybrid clone (viz., CJH 5) for higher seed oil content. The study also found that all the hybrid clones were amenable for biodiesel production due to their acceptable and satisfactory biofuel quality. Hence the hybrid clones with higher seed yield and superiority in chemical properties could be selected for further commercial exploitation to produce biofuel. The research has an excellent application value both in the biofuel sector and in the farming sector. A Jatropha-based multifunctional agroforestry system can act as an alternate and sustainable land-use system, while helping to generate significant income for farmers. It has an excellent application value for creating new start-ups and business enterprises in the form of seed processing, establishment of oil processing plants, and creation of decentralized transesterification plants at the rural level. These establishments will create self-reliance in the green energy sector, thereby extending their significant application value. However, the current research still needs further investigation into the performance of hybrid clones under different agroclimatic zones, coupled with associated physical, chemical, and other biodiesel properties, in order to successfully deploy Jatropha hybrid clones for biodiesel production on a commercial scale.

## Data Availability

The original contributions presented in the study are included in the article/Supplementary Material, and further inquiries can be directed to the corresponding author.

## References

[B1] ASTMD (2002). Biodiesel standard. USA, 6751.

[B22] Association of Official Agricultural Chemists Horwitz W. (1975). Official methods of analysis, 222. Washington, DC: Association of Official Analytical Chemists.

[B2] Azam M. M. Waris A. Nahar N. M. (2005). Prospects and potential of fatty acid methyl esters of some non-traditional seed oils for use as biodiesel in India. Biomass bioenergy 29 (4), 293–302. 10.1016/j.biombioe.2005.05.001

[B3] Bandyopadhay K. R. (2015). Policy Brief on Biofuels promotion in India for transport: Exploring the grey areas. New Delhi: The Energy Research Institute.

[B4] Basha S. D. Sujatha M. (2007). Inter and intra-population variability of *Jatropha curcas* (L.) characterized by RAPD and ISSR markers and development of population-specific SCAR markers. Euphytica 156 (3), 375–386. 10.1007/s10681-007-9387-5

[B5] Bekalu Y. Fekad M. (2020). Review on economic importance of Jatropha apart from its use as a biofuel. Int. J. Res. Dev. 5, 24–33.

[B6] Biabani A. Rafii M. Y. Saleh G. B. Shabanimofrad M. Latif M. A. (2012). Phenotypic and genetic variation of *Jatropha curcas* L populations from different countries. Maydica 57 (2), 164–171.

[B7] Bureau of Indian Standards (2006). Biodiesel specification. New Delhi: Bureau of Indian Standards.

[B8] Chaturvedi O. P. Pandey N. (2001). Genetic divergence in *Bombax ceiba* L. germplasms. Silvae Genet. 50 (3-4), 99–102.

[B9] Chauhan K. C. Gautam S. Thakur I. K. (2005). Genetic variability and correlation studies in seed and seedling traits of neem (*A. indica*). J. Non-Timber For. Prod. 12, 69–75.

[B10] Che Hamzah N. H. Khairuddin N. Siddique B. M. Hassan M. A. (2020). Potential of *Jatropha curcas* L. as biodiesel feedstock in Malaysia: A concise review. Processes 8 (7), 786. 10.3390/pr8070786

[B11] Crabbe E. Nolasco-Hipolito C. Kobayashi G. Sonomoto K. Ishizaki A. (2001). Biodiesel production from crude palm oil and evaluation of butanol extraction and fuel properties. Process Biochem. 37 (1), 65–71. 10.1016/s0032-9592(01)00178-9

[B12] Dehgan Bijan Webster Grady L. (1978). Three new species of jatropha (Euphorbiaceae) from Western Mexico. Madrono 25 (1), 30–39.

[B13] Dewey D. R. Lu K. (1959). A correlation and path‐coefficient analysis of components of crested wheatgrass seed production 1. Agron. J. 51 (9), 515–518. 10.2134/agronj1959.00021962005100090002x

[B14] Divakara B. N. Alur A. S. Tripati S. (2012). Genetic variability and relationship of pod and seed traits in *Pongamia Pinnata* (L.) Pierre., a potential agroforestry tree. Int. J. Plant Prod. 4 (2), 129–142.

[B15] EN (2008). Biodiesel standard. Europe: European Standard Organization. 14214.

[B16] Eriksson Kenneth Estep Don Peter Hansbo Johnson Claes (1995). Introduction to adaptive methods for differential equations. Acta Numer. 4, 105–158. 10.1017/s0962492900002531

[B17] Gawali A. S. Wagh R. S. Sonawane C. J. (2013). Genetic variability in growth and seed traits of *Jatropha curcas* L. germplasm for genetic tree improvement. For. Res. 5, 171. 10.4172/2168-9776.1000171

[B18] Ghosh L. Singh L. (2011). Variation in seed and seedling characters of *Jatropha curcas* L. with varying zones and provenances. Trop. Ecol. 52 (1), 113–122.

[B19] Goodrum J. W. (2002). Volatility and boiling points of biodiesel from vegetable oils and tallow. Biomass Bioenergy 22 (3), 205–211. 10.1016/s0961-9534(01)00074-5

[B20] Goulden C. H. (1936). Methods of statistical analysis. Methods of statistical analysis.

[B21] Gübitz G. M. Georg M. Mittelbach Martin Trabi Manuela (1999). Exploitation of the tropical oil seed plant *Jatropha curcas* L. Bioresour. Technol. 67 (1), 73–82. 10.1016/s0960-8524(99)00069-3

[B23] Kaushik N. Kumar K. Kumar S. Kaushik N. Roy S. (2007). Genetic variability and divergence studies in seed traits and oil content of Jatropha (*Jatropha curcas* L.) accessions. Biomass Bioenergy 31 (7), 497–502. 10.1016/j.biombioe.2007.01.021

[B24] Knothe G. (2002). Structure indices in FA chemistry. How relevant is the iodine value? J. Amer. Oil Chem. Soc. 79 (9), 847–854. 10.1007/s11746-002-0569-4

[B25] Krisnangkura K. (1986). A simple method for estimation of cetane index of vegetable oil. JACOS 63 (4), 56–58.

[B26] Maurya R. Verma S. Gupta A. Singh B. Yadav H. K. (2013). Genetic variability and divergence analyses in *Jatropha curcas* based on floral and yield traits. Genetika 45 (3), 655–666. 10.2298/gensr1303655m

[B27] Ntaganda J. Ndagijimana A. Benimana O. (2014). Characterization of physical and chemical properties of biodiesel produced from *Jatropha curcas* seeds oil cultivated in Rwanda. Sci. J. Energy Eng. 2 (2), 8. 10.11648/j.sjee.20140202.11

[B28] Nzikou J. M. Matos L. Bouanga-Kalou G. Ndangui C. B. Pambou-Tobi N. P. G. Kimbonguila A. Desobry S. (2009). Chemical composition on the seeds and oil of sesame (*Sesamum indicum* L.) grown in Congo-Brazzaville. Adv. J. Food Sci. Technol. 1 (1), 6–11.

[B29] Paramathma M. (2010). Studies on flowering behavior and seed yield of BC4F1 hybrid progenies in Jatropha. Electron. J. Plant Breed. 1 (4), 1066–1069.

[B30] Parthiban K. T. Kirubashankkar R. Paramathma M. Subbulakshmi V. Thiyagarajan P. Vennila S. (2011). Genetic association studies among growth attributes of Jatropha hybrid genetic resources. Int. J. Plant Breed. Genet. 5 (2), 159–167. 10.3923/ijpbg.2011.159.167

[B31] Parthiban K. T. Kumar R. S. Thiyagarajan P. Subbulakshmi V. Vennila S. Rao M. G. (2009). Hybrid progenies in Jatropha–a new development. Curr. Sci., 815–823.

[B32] Reeja P. (2018). Genetic studies in *Jatropha curcas* L. Int. J. Chem. Stud. 6 (4), 3261–3266.

[B33] Sayyed S. Das R. K. Kulkarni K. (2022). Experimental investigation for evaluating the performance and emission characteristics of DICI engine fueled with dual biodiesel-diesel blends of Jatropha, Karanja, Mahua, and Neem. Energy 238, 121787. 10.1016/j.energy.2021.121787

[B34] Seela C. R. Ravi Sankar B. Kishore D. Babu M. V. S. (2019). Experimental analysis on a DI diesel engine with cerium-oxide-added Mahua methyl ester blends. Int. J. Ambient Energy 40 (1), 49–53. 10.1080/01430750.2017.1360203

[B35] Singh B. Singh K. Shukla G. Goel V. L. Pathre U. V. Rahi T. S. (2013). The field performance of some accessions of *Jatropha curcas* L.(Biodiesel Plant) on degraded sodic land in North India. Int. J. green energy 10 (10), 1026–1040. 10.1080/15435075.2012.738336

[B36] Singh S. Prakash A. Chakraborty N. R. Wheeler C. Agarwal P. K. Ghosh A. (2016). Trait selection by path and principal component analysis in *Jatropha curcas* for enhanced oil yield. Industrial Crops Prod. 86, 173–179. 10.1016/j.indcrop.2016.03.047

[B37] Sivaramakrishnan K. Ravikumar P. (2012). Determination of cetane number of biodiesel and its influence on physical properties. ARPN J. Eng. Appl. Sci. 7 (2), 205–211.

[B38] Subashini G. Ibrahim S. M. Paramathma M. Manivannan N. (2014). Studies on genetic variability parameters in backcross population of jatropha (*Jatropha curcas*). Int. J. Trop. Agric. 32 (3/4), 569–572.

[B39] Sujatha M. (2020). Genetic improvement of *Jatropha curcas* (L.) possibilities and prospects.

[B40] Tripathi A. Mishra D. K. Shukla J. K. (2015). Genetic diversity and trait association between growth, yield and seed component of *Jatropha curcas* (L.) source collection from Indian sub-continent. J. Plant Breed. Crop Sci. 7 (5), 143–157.

[B41] Witze A. (2007). Energy: That's oil, folks. Nature 445 (7123), 14–17. 10.1038/445014a 17203036

